# Assessing and Improving Data Integrity in Web-Based Surveys: Comparison of Fraud Detection Systems in a COVID-19 Study

**DOI:** 10.2196/47091

**Published:** 2024-01-12

**Authors:** Stephen Bonett, Willey Lin, Patrina Sexton Topper, James Wolfe, Jesse Golinkoff, Aayushi Deshpande, Antonia Villarruel, José Bauermeister

**Affiliations:** 1 School of Nursing University of Pennsylvania Philadelphia, PA United States; 2 Department of Psychology Ashoka University Sonepat India

**Keywords:** web-based survey, data quality, fraud, survey methodology, COVID-19, survey, fraud detection, Philadelphia, data privacy, data protection, privacy, security, data, information security, data validation, cross-sectional, web-based

## Abstract

**Background:**

Web-based surveys increase access to study participation and improve opportunities to reach diverse populations. However, web-based surveys are vulnerable to data quality threats, including fraudulent entries from automated bots and duplicative submissions. Widely used proprietary tools to identify fraud offer little transparency about the methods used, effectiveness, or representativeness of resulting data sets. Robust, reproducible, and context-specific methods of accurately detecting fraudulent responses are needed to ensure integrity and maximize the value of web-based survey research.

**Objective:**

This study aims to describe a multilayered fraud detection system implemented in a large web-based survey about COVID-19 attitudes, beliefs, and behaviors; examine the agreement between this fraud detection system and a proprietary fraud detection system; and compare the resulting study samples from each of the 2 fraud detection methods.

**Methods:**

The PhillyCEAL Common Survey is a cross-sectional web-based survey that remotely enrolled residents ages 13 years and older to assess how the COVID-19 pandemic impacted individuals, neighborhoods, and communities in Philadelphia, Pennsylvania. Two fraud detection methods are described and compared: (1) a multilayer fraud detection strategy developed by the research team that combined automated validation of response data and real-time verification of study entries by study personnel and (2) the proprietary fraud detection system used by the Qualtrics (Qualtrics) survey platform. Descriptive statistics were computed for the full sample and for responses classified as valid by 2 different fraud detection methods, and classification tables were created to assess agreement between the methods. The impact of fraud detection methods on the distribution of vaccine confidence by racial or ethnic group was assessed.

**Results:**

Of 7950 completed surveys, our multilayer fraud detection system identified 3228 (40.60%) cases as valid, while the Qualtrics fraud detection system identified 4389 (55.21%) cases as valid. The 2 methods showed only “fair” or “minimal” agreement in their classifications (κ=0.25; 95% CI 0.23-0.27). The choice of fraud detection method impacted the distribution of vaccine confidence by racial or ethnic group.

**Conclusions:**

The selection of a fraud detection method can affect the study’s sample composition. The findings of this study, while not conclusive, suggest that a multilayered approach to fraud detection that includes conservative use of automated fraud detection and integration of human review of entries tailored to the study’s specific context and its participants may be warranted for future survey research.

## Introduction

Web-based survey research has become increasingly common in recent years, particularly because of its ability to reach broad populations efficiently and economically [1]. Web-based surveys involve inviting potential respondents to complete questionnaires through digital platforms that manage how questions are presented and how data are collected and stored [2,3]. These research methods have been used in response to the difficulties faced in traditional survey methods (ie, recruiting participants using flyers, newspaper or radio or television advertisements or spreading by word of mouth and collecting data in person using computer-assisted survey instruments or over the phone), especially in reaching underrepresented populations [4,5]. The advantages of web-based surveys include eliminating the requirement for face-to-face interaction, offering flexible access to surveys, removing transportation and logistical barriers, and preserving anonymity. In recent years, COVID-19 pandemic restrictions limited opportunities for in-person research and provided additional justification for researchers to adopt web-based study designs while leveraging social media recruitment methods to reach diverse populations [6-9].

As web-based recruitment and survey methods in health research have become more ubiquitous and refined, so too have methods of web-based research fraud [10,11]. Fraud can manifest in multiple ways. For example, individuals may misrepresent themselves in order to appear eligible for a study or may submit duplicate surveys in order to receive multiple incentive payments. Additionally, fraudulent data may also come from automated operations enacting fraud at a large scale, often referred to as “bots” [11,12]. These methods are often used to target surveys offering participation compensation payments and can be lucrative when aimed at large web-based surveys, even those offering small payments [9,13]. Such fraud poses risks not only to research resources but also, importantly, to the integrity of research findings, as fraudulent data can distort results and undermine data quality. Specifically, fraudulent responses can introduce additional random noise or potentially add systematic bias to the data [14-16].

In response, researchers, companies operating in the digital research space (eg, Qualtrics) [17], and organizations interested in digital data integrity (eg, Google) [18] have developed methods to address fraudulent activity. The research community has crafted recommendations for fraudulent data identification and participant identity verification protocols [13,19,20]. Platforms specializing in web-based survey research such as Qualtrics [17] and Amazon Mechanical Turk [21] have also developed fraud detection features that accompany their services. While these proprietary systems for fraud detection offer a simple, automated approach to improving data quality, little information is available about the mechanisms they use [22]. Fraud detection systems often obscure details about how their validation process functions as an important strategy to protect the integrity of the fraud detection system, making it more difficult for fraudulent participants to circumvent protections. However, obfuscation also introduces questions about how fraud detection algorithms alter study samples and whether they introduce bias into analyses [20].

Little research has compared how fraud detection strategies impact study sample composition or examined their comparative effectiveness in correctly identifying fraud [22-25]. By accurately identifying and removing fraudulent responses to web-based surveys, research can improve data quality and strengthen the overall rigor of their methods. Robust, reproducible, and context-specific methods of accurately detecting fraudulent responses are needed to ensure integrity and maximize the value of web-based survey research. This paper aims to (1) describe the multilayer fraud detection techniques we developed and implemented in a large web-based survey collecting data about attitudes, beliefs, and behaviors related to COVID-19; (2) examine the degree of agreement between our multilayer fraud detection strategy and the proprietary fraud detection system used by Qualtrics; and (3) compare the study samples that resulted when using each of the 2 fraud detection methods.

## Methods

### Study Design

We collected data from November 2021 through February 2022 for the PhillyCEAL Common Survey, a cross-sectional study using a web-based survey to assess how the COVID-19 pandemic and response have impacted individuals, neighborhoods, and communities across the city of Philadelphia, Pennsylvania. The Checklist for Reporting Results on Internet E-Surveys was used to guide the reporting of our methods and results (Multimedia Appendix 1) [26]. The Qualtrics web-based survey platform was used to design the survey and automatically capture responses in a database. The usability and technical functionality of the survey were tested by the study team before launching the survey. Individuals were eligible to participate if they (1) resided within Philadelphia County (coterminous with the city limits) and (2) were at least 13 years of age. We recruited participants through advertisements on social media platforms (ie, Facebook, Instagram, Twitter, and Reddit) and referrals from community partners (including one partner that provided study recruitment materials to individuals via door-to-door canvassing). The recruitment process directed individuals to a voluntary, open web-based survey, where they completed a screener consent form and answered a series of questions to determine eligibility and record basic demographic information. We did not allow participants to change their answers through a back button feature.

Eligible participants were automatically directed to the full study consent form. Following consent, participants were asked to complete a 20-minute questionnaire about their experiences, behaviors, and beliefs about COVID-19, risk of infection, testing, vaccination, treatment, and knowledge and beliefs about COVID-19 clinical trials. Participants completed 1 of 3 slight variations of the survey (ie, the adult survey, the parent survey, and the youth survey), where additional questions or slight changes to wording were used on the parent and youth surveys. Participants completed only 1 of these 3 potential variations (ie, survey groups were mutually exclusive), with participants of any age who reported having minor children completing the parent survey (103 questions), participants ages 25 years or younger and not having minor children completing the youth survey (126 questions), and all other participants completing the adult survey (92 questions). To reduce participant burden, we used adaptive questioning to reduce the number of questions displayed based on their answers. Participants saw an average of 6 questions per page.

Participants were excluded if they did not complete the entire survey (n=2930) or did not provide a residential zip code matching 1 of the 48 zip codes of Philadelphia County (n=647). Participants confirmed eligible and marked as complete by both Qualtrics automation and our manual review of the data were compensated with a US $15 electronic gift card. To protect participant data, the web survey data were downloaded to a secure university server, deidentified by replacing contact information with unique IDs, and stored in a restricted folder with password protection.

### Ethical Considerations

All participants completed an informed consent process before proceeding to the survey. Ethics approval was obtained from the institutional review board at the University of Pennsylvania (protocol 848650).

### Preliminary Fraud Protection

Given the prevalence of fraud and duplicate responses in web-based survey research, we used several strategies to prevent fraudulent participants from accessing and completing the survey, serving as a baseline level of fraud protection for both our multilayer and the Qualtrics detection methods. Our preliminary line of defense against fraud was targeted toward nonhuman interferences such as bots. To proceed with the survey, all respondents had to pass a built-in Turing test provided by Qualtrics using Google’s reCAPTCHA (version 2) antifraud technology [27]. Depending on the respondent’s on-device saved data such as browser cookies, they either had to click a checkbox or solve a simple image challenge to pass the reCAPTCHA (version 2) test.

Since sophisticated bots can trick the reCAPTCHA (version 2) test [28], we added a honeypot question as a second line of defense against bots. Honeypots are survey questions hidden from rendering on the screen using custom JavaScript code [11]. They are, therefore, invisible to human respondents but accessible to bots that do not rely on what is rendered on screen. Since the honeypot is not visible to human respondents, any responses to the honeypot would immediately disqualify the entire survey response and end the survey.

We also created unique URLs for each recruitment source and advertisement campaign for the study. The unique URLs enabled us to identify the origin (recruitment source and ad campaign) of each survey response. This allowed us to individually monitor and suspend links that became the target of fraudulent survey responses.

### Multilayer Fraud Detection Methods

#### Real-Time Exclusion of Fraudulent Responses

We implemented a suite near real-time data verification procedures to assess the veracity of data as they were collected, including techniques based on recommendations from prior research as well as several manual checks that were developed specifically for this study. A research team member would individually inspect submitted responses and label responses as fraudulent using the following criteria: (1) participants were asked to provide their residential address and the colloquial name of their neighborhood. Responses were marked as fraud if the neighborhood name provided did not match a standard Philadelphia neighborhood name corresponding to the residential address provided or any adjacent neighborhood; (2) the residential address provided did not match an existing address in Philadelphia County [20,23,29]; (3) the survey had the same start times and stop times plus or minus 1 minute as 2 or more other submitted surveys (rapid survey submission) [11,29]; (4) the respondent’s email address matched a previously enrolled participant’s email address [11,20]; (5) the zip code provided as part of the residential address was nonstandard (ie, a post office box code or a unique code) [20,23]; (6) the residential address had already been reported by at least 2 other respondents [20,23]; or (7) the URL from which the response was referred did not match any of the URL links distributed by the study team during recruitment. Responses labeled as fraud during real-time validation were not eligible to receive survey compensation.

#### Automated Post Hoc Identification of Fraudulent Responses

We developed a set of automated post hoc techniques designed to detect fraud that our real-time procedures may not have captured. Three criteria were developed for this post hoc fraud identification based on recommendations from prior research [11,20,22,23,29-32]. Since these criteria only identify suspicious entries and do not definitively prove that an entry is fraudulent, responses were labeled as fraud only if they satisfied 2 or more criteria. We settled on using a threshold of 2 criteria (rather than 1 or 3) in order to balance concerns about the potential of each of our 3 criteria to incorrectly label a participant as fraud with the necessity to exclude causes that showed strong evidence of fraud. The criteria were as follows:

1. In response to a free text item at the end of the survey soliciting additional comments or questions from the participant, the submitted survey included text that was identical to text submitted by other respondents. We considered a free text response an identical match if it was among free text entries of 1 word or greater that were repeated 100 or more times, free text entries of 2 words or greater that were repeated 10 or more times, or free text entries of 3 words or greater that were repeated 3 or more times (see Table S1 in Multimedia Appendix 2 for list of unique text strings excluded and their frequency in the full set of responses);
2. The IP address of a response belonged to a virtual private network or data center or originated from outside the United States, as determined by using a security service for proxy and virtual private network detection and IP location information [33]; and
3. Responses provided in the main survey were inconsistent with responses to the same items in the screener for one or more key items that would not be expected to be variable (ie, age, zip code, number of adults living at home with the participant, number of minors living at home with the participant, Hispanic or Latinx ethnicity, “Have you ever been tested for COVID-19?,” and “Have you received at least 1 dose of the COVID-19 vaccine?”).

### Qualtrics Fraud Detection Methods

Qualtrics is a widely used web-based survey platform that allows users to create surveys with complex flow logic and customizable visual design. Qualtrics surveys are easily optimized for use on mobile devices and can display a wide variety of question types on both computer and mobile phone interfaces. Another key strength of the Qualtrics platform is its integration of 1-click translation, allowing users to quickly switch between various languages. This was crucial for our study, which recruited participants from diverse populations across Philadelphia and was available in English, Spanish, and Mandarin. In addition to these valuable features, Qualtrics also offers tools for detecting fraudulent survey responses. This automated and user-friendly system for fraud detection has the potential to help researchers improve data quality in their web-based surveys. Given the lack of research exploring how these consumer tools compare to existing published protocols for fraud detection, we sought to compare our multilayer fraud detection methods to the system used by Qualtrics.

The Qualtrics fraud detection system relies on Google’s reCAPTCHA (version 3) and Imperium’s RelevantID antifraud technologies. Both tools rely on proprietary machine learning models that analyze passive and behavioral data, browser interactions, and respondent metadata to identify abuse and fraud [18,34,35]. Unlike the reCAPTCHA (version 2) test respondents had to solve at the start of the survey, bot detection using reCAPTCHA (version 3) does not present respondents with an image challenge nor block respondents and bots from proceeding with the survey. Instead, it returns a score (Q_RecaptchaScore) between 0.0 and 1.0 that Qualtrics records as part of the survey response. We used the recommended 0.5 score as the threshold for fraud, where a score under 0.5 is deemed likely to be a bot [17,18].

Like reCAPTCHA (version 3), RelevantID does not prevent bots from completing the survey. Instead, it attaches a score (Q_RelevantIDFraudScore) between 0 and 130 to each survey response. We followed Qualtrics’ recommendation in interpreting a score ≥30 as fraudulent and likely a bot [17]. In addition to bot detection, RelevantID identifies duplicate responses through digital fingerprinting and proprietary detection algorithms [34]. Qualtrics then attaches another score (Q_RelevantIDDuplicateScore) between 0 and 100 to the survey response. We followed the suggested score threshold where any score ≥75 is considered a duplicate [17].

### Statistical Analysis

#### Agreement and Comparative Performance

The classification tables were created to display the degree of agreement between the 2 fraud detection methods for the full sample and for each of the 3 survey-type categories (ie, adult, parent, and youth).

#### Impact of Fraud Detection Method on Sample Characteristics

Descriptive statistics were computed for the full sample of responses, the subset classified as valid by our multilayer fraud detection method, and the subset classified as valid by the Qualtrics fraud detection method. As these 3 sets of responses are not mutually exclusive, we did not directly compare them statistically.

To test for differences between fraudulent and valid responses as classified by each fraud detection method, statistical comparisons were conducted for key study variables between the mutually exclusive sets of responses classified as fraudulent or valid within each method. Specifically, we used chi-square tests for categorical variables, 2-tailed *t* tests for normally distributed continuous variables, and Mann-Whitney *U* tests for continuous variables that were not normally distributed. The results of these analyses are presented in Table S2 in Multimedia Appendix 2 for the multilayer method and Table S3 in Multimedia Appendix 2 for Qualtrics.

To assess the degree to which the 2 fraud detection methods would impact the distribution of a key study variable, the point estimate and 95% CI were calculated for vaccine confidence by racial or ethnic group for each fraud detection method and for the entire sample without any fraud mitigation.

#### Variations in Survey Responses During Study Period

A time-series plot was created to show the cumulative responses to the study survey over time and their fraud classification by each of the 2 fraud detection methods. This plot highlights the periods in which social media recruitment campaigns are active and can also shed light on how the 2 fraud detection methods diverge in their classification of responses during different periods of high survey response. Additionally, we present a time-series plot showing the proportion of responses classified as fraud across the study period, including smooth locally weighted smoothing lines to visualize the trends over time. All analyses were performed with R (version 4.1.0; R Foundation for Statistical Computing).

## Results

### Multilayer Fraud Detection Methods

**Figure 1:**
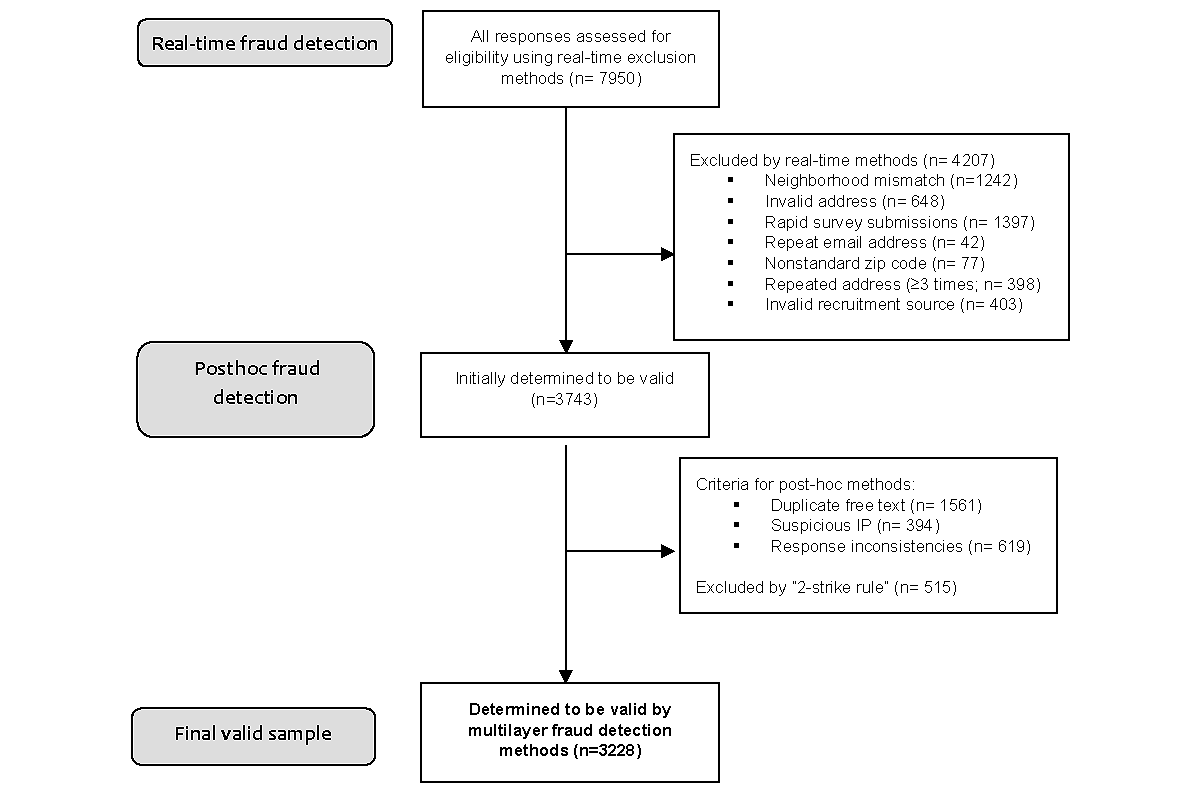
Flowchart of responses through the multilayer fraud detection methods developed and implemented in this study.

**Figure 2:**
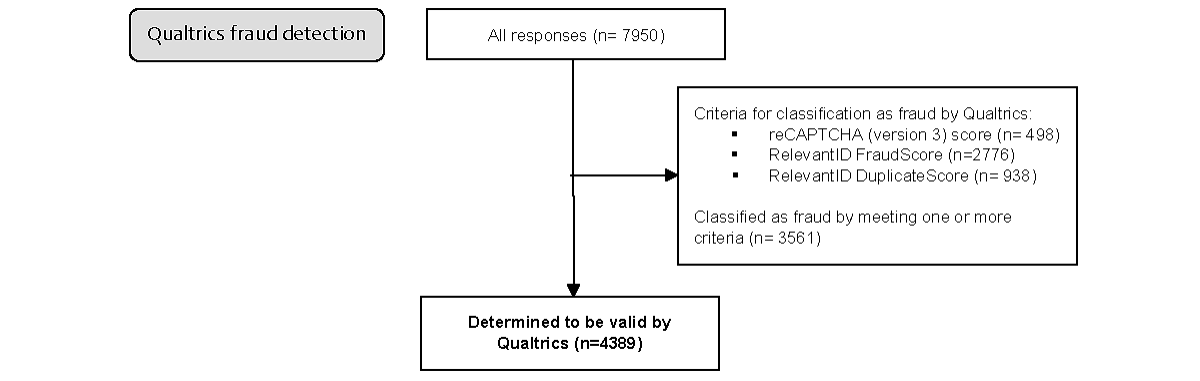
Flowchart of responses through the alternative fraud detection methods provided by the Qualtrics survey platform.

A total of 7950 completed survey responses were received. See Figures 1 and 2 for an overview of fraud detection results from our multilayer fraud detection methods. Using the real-time exclusion criteria of the multilayer fraud detection method, 4207 (52.92%) entries were classified as fraud. Of those classified as fraud, 1242 (29.52%) reported a neighborhood name that did not match their residential address, 648 (15.4%) provided an invalid residential address, 1397 (33.21%) displayed rapid survey submission, 42 (1%) used a repeated email address, 77 (1.83%) reported a nonstandard zip code, 398 (9.46%) reported a residential address that was used more than twice, and 403 (9.58%) did not have a valid recruitment URL. After the real-time exclusion, 3743 (47.08%) cases remained classified as valid.

Our automated post hoc fraud detection criteria identified additional cases as fraud. Of the remaining 3743 initially valid cases, 1561 (41.70%) cases had a duplicate response in the free text entry item, 394 (10.53%) cases had an IP address from outside the United States or from a virtual private network, and 619 (16.54%) had inconsistencies between the screener and main survey on at least 1 key item. Using our “2-strike” rule, we classified an additional 515 (13.76%) responses as fraud for meeting at least 2 of the above criteria. Thus, our multilayer fraud detection strategy classified a total of 4722 (59.40%) entries as fraud and 3228 (40.60%) entries as valid.

### Qualtrics Fraud Detection Methods

The Qualtrics fraud detection methods identified 498 (6.26%) cases that failed reCAPTCHA (version 3), 2776 (34.92%) cases as fraud by the RelevantID FraudScore, and 938 (11.80%) cases as duplicates by the RelevantID DuplicateScore. The Qualtrics fraud detection strategy classified a total of 3561 (44.79%) entries as fraud (ie, meeting one or more of the 3 criteria above) and 4389 (55.21%) entries as valid.

### Agreement and Comparative Performance

Table 1 presents confusion matrices showing the degree of agreement between our multilayer fraud detection method and the Qualtrics fraud detection method for the full sample and each survey-type category. The interrater reliability indicated “fair” or “minimal” agreement between the 2 methods for the full sample (κ=0.25; 95% CI 0.23-0.27), “moderate” or “weak” agreement for the adult (κ=0.48; 95% CI 0.43-0.53) and youth (κ=0.50; 95% CI 0.43-0.58) surveys, and “slight” or “none” agreement for the parent survey (κ=0.13; 95% CI 0.10-0.15) [36,37].

**Table 1:** Confusion matrix and interrater reliability (κ) between our multilayer fraud detection system and the Qualtrics fraud detection system for the full sample, only adult surveys, only parent surveys, and only youth surveys.

		Fraud (multilayer)	Valid (multilayer)
**Full sample^a^**			
	Fraud (Qualtrics)	*2627^b^*	934^c^
	Valid (Qualtrics)	2095^c^	*2294*
**Adult survey^d^**			
	Fraud (Qualtrics)	*299*	166^c^
	Valid (Qualtrics)	174^c^	*904*
**Parent survey^e^**			
	Fraud (Qualtrics)	*2184*	710^c^
	Valid (Qualtrics)	1848^c^	*1102*
**Youth survey^f^**			
	Fraud (Qualtrics)	*144*	58^c^
	Valid (Qualtrics)	73^c^	*288*

^a^κ=0.25; 95% CI 0.23-0.27.

^b^Values in italics represent agreement between the 2 methods.

^c^Values represent disagreement between the 2 methods.

^d^κ=0.48; 95% CI 0.43-0.53.

^e^κ=0.13; 95% CI 0.10-0.15.

^f^κ=0.50; 95% CI 0.43-0.58.

We conducted sensitivity analyses to assess the impact of choosing a “2-strike rule” for our post hoc fraud detection rather than a “1-strike rule” or a “3-strike rule.” Compared to the “2-strike rule,” which resulted in 515 additional cases being classified as fraud during the post hoc phase of fraud detection, the “1-strike rule” would have classified 2047 additional cases as fraud, and the “3-strike rule” would have classified 12 additional cases as fraud. In terms of agreement with Qualtrics’ fraud detection methods, the “1-strike rule” would have resulted in a κ of 0.20 (95% CI 0.19-0.22) for the full sample, and the “3-strike rule” would have resulted in a κ of 0.24 (95% CI 0.22-0.26) for the full sample.

Additionally, we explored how the 2 fraud detection strategies compared in their ability to classify cases with validated email addresses as valid entries. Validated email addresses were defined as email addresses ending in “.edu” or “.gov,” indicating an institutional affiliation. Of the 168 cases with validated emails, the multilayer fraud detection system correctly classified 166 (98.81%) as valid, while the Qualtrics fraud detection system correctly classified only 126 (75%) as valid.

### Impact of Fraud Detection Method on Sample Characteristics

Decisions about which fraud detection strategies to use can impact the results of web-based survey research. Table 2 presents the descriptive statistics for sociodemographic variables, survey metric variables, and key study outcome variables on 3 versions of the data set: the full data set with no fraud detection (n=7950), the cases identified as valid by our multilayer fraud detection methods (n=3228), and the cases identified as valid by the Qualtrics fraud detection methods (n=4389). As these sets are not mutually exclusive, we cannot compare them directly; however, there are clear differences in the distributions of many study variables between the 3 sets. When comparing entries classified as fraud to those classified as valid for each of the 2 fraud detection methods (ie, mutually exclusive sets), all study variables, except for lifetime COVID-19 testing for the multilayer fraud detection, were found to be significantly different for both methods (Tables S2 and S3 in Multimedia Appendix 2).

Table 3 showcases in detail how a key variable of interest to researchers may be affected by using different fraud detection methods. In this data set, vaccine confidence among White respondents was greater when using our multilayer fraud detection (µ=0.867; 95% CI 0.851-0.882) when compared to Qualtrics fraud detection (µ=0.782; 95% CI 0.766-0.798). A similar pattern is seen for Hispanic or Latinx respondents and Black or African American respondents.

**Table 2:** Demographics, survey metrics, and key study responses in overall sample, multilayer valid set, and Qualtrics valid set.

							Full sample (N=7950)				Multilayer valid set (n=3228)				Qualtrics valid set (n=4389)
**Demographics**															
	Age (years), mean (SD)					35.54 (9.70)				38.09 (12.15)				37.01 (10.81)	
	**Race or ethnicity, n (%)**														
		Hispanic or Latinx			1188 (14.9)				254 (7.9)				571 (13)		
		**Non-Hispanic**													
			American Indian or Alaska Native	135 (1.7)				7 (0.2)				34 (0.8)			
			Asian	311 (3.9)				219 (6.8)				221 (5)			
			Black or African American	1856 (23.3)				728 (22.6)				853 (19.4)			
			Native Hawaiian or Pacific Islander	42 (0.5)				11 (0.3)				10 (0.2)			
			White	4272 (53.7)				1889 (58.5)				2600 (59.2)			
			Multiracial or others	146 (1.8)				120 (3.7)				100 (2.3)			
	**Gender, n (%)**														
		Woman			4253 (53.5)				2028 (62.8)				2645 (60.3)		
		Man			3571 (44.9)				1108 (34.3)				1663 (37.9)		
		Transgender or gender diverse			105 (1.3)				76 (2.4)				64 (1.5)		
		Prefer not to answer			21 (0.3)				16 (0.5)				17 (0.4)		
	**Sexual orientation, n (%)**														
		Bisexual			361 (4.5)				242 (7.5)				262 (6)		
		Gay			231 (2.9)				101 (3.1)				147 (3.3)		
		Lesbian			142 (1.8)				69 (2.1)				65 (1.5)		
		Straight (ie, not gay, lesbian, or bisexual)			7039 (88.5)				2682 (83.1)				3791 (86.4)		
		Others			94 (1.2)				84 (2.6)				71 (1.6)		
		Prefer not to answer			83 (1)				50 (1.5)				53 (1.2)		
	**Education, n (%)**														
		Less than high school			215 (2.7)				49 (1.5)				65 (1.5)		
		High school or equivalent			1013 (12.7)				299 (9.3)				479 (10.9)		
		Some college			1890 (23.8)				579 (17.9)				964 (22)		
		College graduate			3935 (49.5)				1672 (51.8)				2253 (51.3)		
		Graduate degree			882 (11.1)				620 (19.2)				617 (14.1)		
		Prefer not to answer			15 (0.2)				9 (0.3)				11 (0.3)		
	**Survey type, n (%)**														
		Adult			1543 (19.4)				1070 (33.1)				1078 (24.6)		
		Parent			5844 (73.5)				1812 (56.1)				2950 (67.2)		
		Youth			563 (7.1)				346 (10.7)				361 (8.2)		
**Survey metrics**															
	Survey duration (minutes), median (IQR)					23.46 (18.38-38.10)				22.02 (18.13-32.57)				22.82 (18.52-35.13)	
	User language=Spanish, n (%)					127 (1.6)				22 (0.7)				53 (1.2)	
**Key study variables**															
	Ever tested for COVID-19=yes, n (%)					6968 (87.6)				2840 (88)				3903 (88.9)	
	**Ever COVID-19–positive, n (%)**														
		No			5836 (83)				2498 (87.3)				3294 (83.8)		
		Yes			1098 (15.6)				352 (12.3)				593 (15.1)		
		Do not know or prefer not to answer			95 (1.3)				10 (0.3)				42 (1.1)		
		N/A^a^			921 (11.6)				368 (11.4)				460 (10.5)		
	**COVID-19 vaccination status, n (%)**														
		No, have not gotten the vaccine			458 (5.8)				66 (2)				215 (4.9)		
		Yes, first dose of 2-dose vaccine			765 (9.6)				137 (4.2)				287 (6.5)		
		Yes, both doses of 2-dose vaccine			5518 (69.4)				2815 (87.2)				3465 (78.9)		
		Yes, 1-dose vaccine			933 (11.7)				166 (5.1)				295 (6.7)		
		Yes, more than 2 doses of a vaccine			196 (2.5)				32 (1)				111 (2.5)		
		Do not know or prefer not to answer			80 (1.1)				12 (0.4)				16 (0.3)		
	**COVID-19 vaccine confidence, n (%)**														
		Not at all confident			250 (3.1)				43 (1.3)				144 (3.3)		
		Not too confident			1364 (17.2)				399 (12.4)				732 (16.7)		
		Somewhat confident			3041 (38.3)				1055 (32.7)				1558 (35.5)		
		Very confident			3184 (40.1)				1696 (52.5)				1898 (43.2)		
		Do not know or prefer not to answer			111 (1.4)				35 (1)				57 (1.3)		

^a^N/A: not applicable.

**Table 3:** COVID-19 vaccine confidence (somewhat confident or very confident) grouped by race compared across the 2 fraud detection methods.

Race or ethnicity		Multilayer valid set (n=3228)				Qualtrics valid set (n=4389)		
		n (%)	Mean (SD)	95% CI	n (%)		Mean (SD)	95% CI
Hispanic or Latinx		254 (7.9)	0.87 (0.34)	0.82-0.91	571 (13)		0.80 (0.40)	0.77-0.83
**Non-Hispanic**								
	American Indian or Alaska Native	7 (0.2)	1.00 (0.00)	1.00-1.00	34 (0.8)		0.79 (0.41)	0.66-0.93
	Asian	219 (6.8)	0.92 (0.28)	0.88-0.95	221 (5)		0.90 (0.31)	0.86-0.94
	Black or African American	728 (22.6)	0.80 (0.40)	0.77-0.83	853 (19.4)		0.77 (0.42)	0.74-0.80
	Native Hawaiian or Pacific Islander	11 (0.3)	1.00 (0.00)	1.00-1.00	10 (0.2)		0.80 (0.42)	0.54-1.06
	White	1889 (58.5)	0.87 (0.34)	0.85-0.88	2600 (59.2)		0.78 (0.41)	0.77-0.80
	Multiracial or others	120 (3.7)	0.76 (0.43)	0.68-0.84	100 (2.3)		0.77 (0.42)	0.69-0.85

### Variations in Survey Responses During Study Period

**Figure 3:**
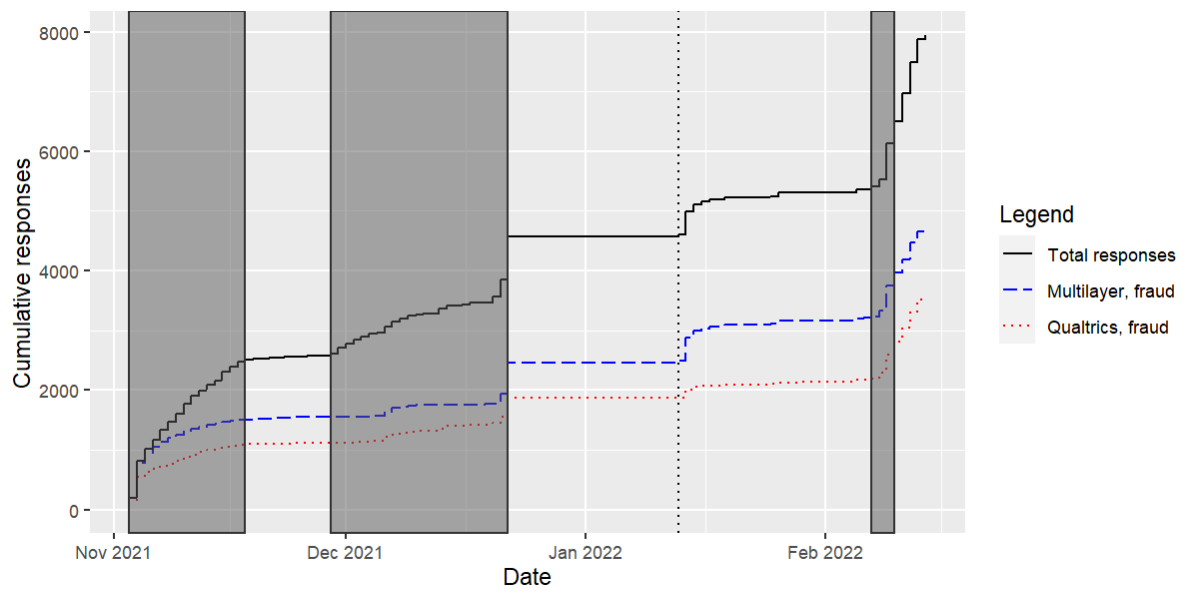
Fraud detection by multilayer fraud detection system and Qualtrics fraud detection system during the study period (November 2021 to February 2022). Highlighted regions indicate periods when social media campaigns were active (November 3-18, 2021; November 29-December 22, 2021; and February 7-10, 2022. Data collection was paused during an extended winter break from December 23, 2021, until January 12, 2022. The dotted vertical line represents January 12, 2022, when data collection was resumed.

**Figure 4:**
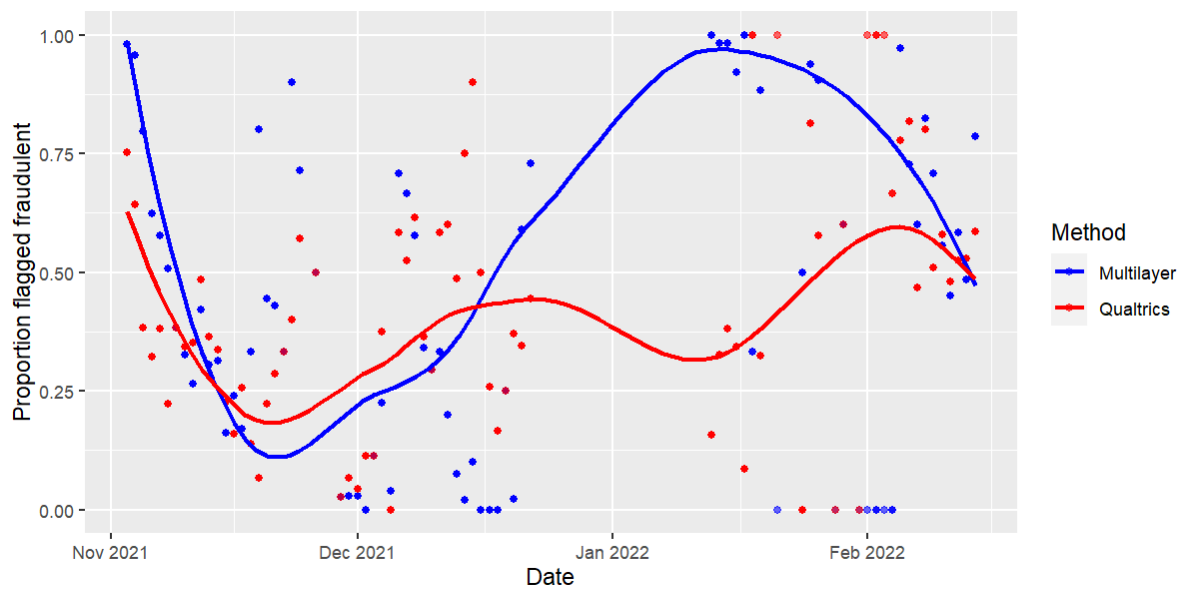
Proportion of responses classified as fraud by multilayer fraud detection system and Qualtrics fraud detection system during the study period (November 2021 to February 2022). Smooth locally weighted smoothing lines are included to help visualize the trends over time.

The response rate varied throughout the study and was correlated to several social media advertising campaigns and an extended holiday break where no responses were accepted. Figure 3 shows the cumulative number of responses over time, differentiated by fraud detection method and fraud classification. Time periods when social media advertising campaigns were active are highlighted on these plots. Responses tend to increase during social media campaigns. Notably, between January 13 and February 7, 2022, no social media campaign was active, yet a significant number of responses were received (n=766). These responses were largely classified as fraud by our multilayer fraud detection method (n=716, 93.47% classified as fraud) but were often classified as valid by the Qualtrics fraud detection method (n=296, 38.64% classified as fraud). Figure 4 shows the proportion of responses classified as fraud across the study period. The comparative fraud detection between the 2 methods was similar during the first half of the study period (November to December 2021), while the multilayer fraud detection method consistently identified a higher proportion of responses as fraud during the later portion of the study period (January to February 2022).

## Discussion

### Principal Findings

Our multilayer fraud detection methods identified a substantial number of fraudulent cases. However, when comparing our fraud detection methods to proprietary fraud detection systems provided by web-based survey software, we saw low levels of agreement between the 2 methods. Our results highlight how the choice of fraud detection method can alter the distribution of key study variables.

Both our multilayer fraud detection methods and the Qualtrics fraud detection system identified significant levels of fraud; however, the 2 methods differed in which cases they identified as fraudulent and in which they identified as valid. Notably, these differences were most pronounced for participants who administered the survey specifically for parents. It is possible that fraudulent participants made assumptions about eligibility or compensation (eg, parents are a more specific demographic that may be of interest to researchers, and thus, fraudulent entries that claim to be parents may be more likely to screen as eligible and receive compensation) and responded to eligibility questions in ways that guided them to the parent survey. With a greater number, and perhaps a greater variety, of fraudulent participants, we then may have seen greater variability in the 2 methods’ ability to consistently identify the fraud.

The disagreement between our multilayer fraud detection and the Qualtrics fraud detection suggests that there are important differences in the methodologies being used by the 2 systems, which resulted in differences in classification. To fully understand and compare the relative performance of the 2 systems, detailed information is needed about the methods used by the Qualtrics fraud detection system. It is likely that the features of the RelevantID proprietary fraud detection method used by Qualtrics are intentionally obfuscated to prevent fraudulent participants from undermining its effectiveness. The trade-off for this black box tactic is that researchers who use the Qualtrics platform cannot ascertain how Qualtrics’ fraud detection algorithms function and how these methods compare to alternative fraud detection strategies. There is an inherent tension between transparency (ie, publishing the features of a fraud detection method improves scientific rigor) and defending against fraud (ie, making comprehensive information about a fraud detection method available may enable fraudulent participants to avoid detection) [22]. Additional research is needed to evaluate the effectiveness of proprietary fraud detection systems and compare them to published techniques used by researchers.

For both fraud detection methods, the cases identified as fraud differed in nonrandom ways from the cases classified as valid on key study variables. These differences could have implications for the interpretation of study results; if legitimate survey participants are classified as fraudulent, critical data are lost, and potential bias could be introduced. In addition, many automated fraud detection tools turn to proprietary machine learning data and predictive modeling for fraud detection [22,38]. This could disproportionately affect those with low literacy or barriers to internet access, as fraud detection tools may be more likely to flag them as fraudulent [19]. Given the existing digital divide across racial or ethnic groups [39,40], this may result in the further exclusion of racial or ethnic minorities in research if they are more likely to use older technologies and shared devices at home or in public spaces, such as computers at libraries or community centers (which may trigger the threshold for fraud due to user behavior that is atypical of a single-user device). One approach to overcoming this challenge is to integrate manual inspection of survey entries in place of or in addition to automatic processes that could result in bias [41,42]. However, human inspection of each survey entry can be time-consuming, more variable than automated processes, and could also result in bias. Future work should aim to better characterize subgroups that may be disproportionately flagged by fraud detection systems and develop ensemble approaches that integrate manual and automatic fraud detection while balancing fraud detection accuracy with protections against excluding valid participants.

While overly sensitive fraud detection could result in bias, fraud detection methods that are not sensitive enough to detect fraudulent entries could also add random noise or systematic bias to the data and threaten the integrity of the research [14-16]. It is important to note that we do not have insight into fraudulent participants’ techniques for responding to survey questions. Fraudulent participants may deliberately select specific demographic options (characteristics they believe will be more likely to result in their entry into the study), randomly select their responses, or use some combination of those techniques [9]. Additionally, rapid developments in machine learning and artificial intelligence have increasingly allowed bots to mimic human behavior [11,22], which could contribute to the seemingly human selection of responses on these surveys, including entries into free text fields [43]. Regardless, this analysis demonstrated the importance of developing study-specific fraud detection methods to supplant or supplement the proprietary fraud detection methods of web-based survey platforms.

Another point of note is the decreased effectiveness of fraud detection tools in determining user legitimacy, as major technology companies take increasing measures to protect user privacy. For example, it is common for fraud detection tools to rely on device fingerprinting and browser cookies to help determine the legitimacy of an individual [44]. While these 2 methods are regularly used by advertisers and marketers to track individuals and deliver targeted advertisements, they also provide a way for fraud detection tools to flag known bad actors and differentiate between legitimate and fraudulent responses. However, the invasive and comprehensive nature of device and browser fingerprinting has raised privacy concerns from users and privacy advocates alike [45,46]. Technology companies, such as Apple, Mozilla, and Brave, have in turn introduced measures to hide users’ identities and activity in a bid to protect user privacy. For instance, Apple’s Safari browser on the macOS desktop operating system now strips all unique identifiers from a user’s device profile, so they appear no different from millions of other Safari users [47]. These privacy-protecting measures, while helpful in safeguarding an individual’s digital presence, make it more difficult for fraud detection tools to differentiate between a legitimate human and a bot. This could partially explain the discrepancy we found between the fraud detection by Qualtrics using reCAPTCHA and RelevantID and our multilayer fraud detection.

Without a method to make a conclusive determination regarding which entries are truly fraudulent and which entries are genuinely valid, it is difficult to compare the relative performance of our multilayer fraud detection methods with the Qualtrics fraud detection methods. However, several pieces of evidence suggest that our fraud detection methods have advantages over Qualtrics in this study context. First, we saw that for email addresses that had an institutional affiliation (ie, “.edu” or “.gov,” which require identity confirmation and cannot be generated en masse) and thus were presumed to be valid, our fraud detection methods correctly validated 98% (n=166) of cases. In comparison, Qualtrics only validated 75% (n=126) of cases. Second, we saw an unusually large discrepancy between the 2 fraud detection methods during a period when the survey link was open, but no advertising or recruitment had recently been active. During this time when we did not expect to receive legitimate responses, we received hundreds of responses that were largely classified as fraud by our fraud detection methods but were generally classified as valid by the Qualtrics system. While it is possible that valid participants were still able to find and access this survey in the absence of active recruiting, we believe this pattern is evidence of noneligible actors using automated systems in an attempt to gain additional compensation payments from the survey. Taken together, these 2 observations are indirect evidence that our multilayer fraud detection method may have better specificity (ie, can correctly identify valid entries) as well as better sensitivity (ie, can accurately detect fraudulent entries) when compared to the Qualtrics system in this study. While we are unable to conclude whether the approach we developed for this study is more or less accurate in identifying fraud when compared to the system used by Qualtrics, we believe these pieces of indirect evidence suggest that using an automated system, such as the one available through Qualtrics, alone may be suboptimal. A multilayered approach was recommended to fraud detection that includes conservative use of automated fraud detection and integration of human review of entries that is tailored to the study’s specific context and its participants.

### Limitations

This study is subject to several limitations. First, our comparison of fraud detection methods is limited by the fact that we are unable to definitively determine which entries are valid and which are fraudulent. We selected fraud detection criteria specifically intended to identify repeat respondents (eg, multiple responses providing identical information), fraudulent submissions from outside the Philadelphia region (eg, location verification using IP addresses), and submissions from bots or bot-assisted fraudulent participants (eg, requiring responses that would be difficult to generate via algorithm such as local, colloquial neighborhood names). Applying these criteria may still have resulted in the inclusion of illegitimate responses and the exclusion of legitimate ones. Second, because we cannot know for certain the true fraud status of participants, we are unable to calculate metrics like precision and recall for the fraud detection methods. Future research should aim to establish gold-standard indicators for fraud that could then be used to directly compare the efficacy of the different methods for fraud detection. Third, without knowing how Qualtrics detects fraud, we are unable to determine which components of our strategy may overlap with the Qualtrics strategy. This limits the conclusions we can draw about the comparative effectiveness of these fraud detection methods. Fourth, while we choose to compare our fraud detection methods with the automated systems used by the Qualtrics platform, we believe that similar comparisons and research are also needed with other proprietary fraud detection systems.

### Recommendations

The following recommendations are offered for improving data integrity in web-based survey research based on the findings from this study:

- Use a multilayered approach to fraud detection that combines different techniques like bot detection, location verification, consistency checks, and manual review. Relying solely on one method may miss certain types of fraud.
- Carefully evaluate proprietary fraud detection systems and request details on their methodology if possible. Black box methods make it difficult to fully assess their impact on sample composition.
- Avoid overly strict fraud detection rules that may disproportionately exclude valid respondents from vulnerable groups. Balance rigor with inclusion.
- Continuously monitor survey responses over time to identify changes in fraud patterns that may require adjustments to detection methods.

### Conclusions

Web-based research and recruitment through social media platforms offer powerful flexibility for researchers to collect large, diverse samples. Web-based surveys, however, are vulnerable to low-quality data from fraud and duplicate entries. Researchers must actively design their web-based studies with this vulnerability in mind and adopt active and adaptable methods of detecting and responding to fraudulent survey responses. Automated, proprietary fraud detection systems offered by web-based survey software may be an important tool in combating fraud, but additional research is needed to evaluate their effectiveness. Human verification of survey entries, while time-consuming, can add another layer of protection and enhance the rigor of web-based survey research. We believe a multilayered strategy that includes a combination of automated fraud detection tools, data enrichment, and human intelligence is the best approach for combating fraud.

## Appendix

Multimedia Appendix 1 Checklist for Reporting Results of Internet E-Surveys (CHERRIES).

Multimedia Appendix 2 Fraud detection analysis.

## Data Availability

The data sets generated and analyzed during this study are available from the corresponding author on reasonable request.
