# Correction: Assessing and Improving Data Integrity in Web-Based Surveys: Comparison of Fraud Detection Systems in a COVID-19 Study

**DOI:** 10.2196/76462

**Published:** 2025-04-30

**Authors:** Stephen Bonett, Willey Lin, Patrina Sexton Topper, James Wolfe, Jesse Golinkoff, Aayushi Deshpande, Antonia Villarruel, José Bauermeister

**Affiliations:** 1 School of Nursing University of Pennsylvania Philadelphia, PA United States; 2 Department of Psychology Ashoka University Sonepat India

In “Assessing and improving data integrity in web-based surveys: Comparison of fraud detection systems in a COVID-19 study” (JMIR Form Res 2024;8:e47091) the authors made one change.

The Acknowledgments section has been updated from:

This work was supported by funding from the National Institutes of Health Agreement OT2HL16156 as part of the Community Engagement Alliance Against COVID-19 Disparities, PhillyCEAL.

to:

This research was, in part, funded by the National Institutes of Health (NIH) Agreement OT2HL158287. The views and conclusions contained in this document are those of the authors and should not be interpreted as representing the official policies, either expressed or implied, of the NIH.

The correction will appear in the online version of the paper on the JMIR Publications website, together with the publication of this correction notice. Because this was made after submission to PubMed, PubMed Central, and other full-text repositories, the corrected article has also been resubmitted to those repositories.

