# Supplementary material for: Assessing and Improving Data Integrity in Web-Based Surveys: Comparison of Fraud Detection Systems in a COVID-19 Study
Source: JMIR Form Res. 2024 Jan 12;8:e47091. doi: 10.2196/47091 (PMC10818231; doi:10.2196/47091)
Supplement: Multimedia Appendix 2 [file formative_v8i1e47091_app2.docx]

**Multimedia Appendix 2**

**Table S1.** Commonly repeated free text entries to the final survey question (“Do you have any questions or comments regarding this survey that you would like us to know about?”). Entries with the words and phrases listed below were flagged as meeting the commonly repeated free text entries criteria for the post-hoc fraud analysis, and thus were given a “strike”; two strikes resulted in being labeled as fraud.

| \| Word or Phrase \| Number of occurrences \| \| --- \| --- \| \| ***One word (cut-off: 100 or more occurrences)*** \| ***-*** \| \| no \| 183 \| \| No \| 2427 \| \| None \| 180 \| \| ***Two words (cut-off: 10 or more occurrences)*** \| ***-*** \| \| All good \| 10 \| \| ***Three or more words (cut-off: 3 or more occurrences)*** \| ***-*** \| \| All been good \| 3 \| \| Hope to keep abreast of your latest information \| 5 \| \| I don't have \| 4 \| \| I hope more and more people get vaccinated so that the society will be better and better \| 5 \| \| I hope the survey will be shorter \| 3 \| \| I hope this epidemic will end as soon as possible. I hope the government can promote the role of vaccines, so that more people can be vaccinated, vaccinated, overcome this difficulty as soon as possible, so that everyone's life back to normal! \| 9 \| \| I hope to pay more attention to the epidemic and more people get vaccinated, so that the epidemic can be effectively controlled \| 6 \| \| It was a perfect survey. \| 3 \| \| No thank you \| 4 \| \| No, I don't \| 5 \| \| No, thank you. \| 4 \| \| No, there aren't \| 4 \| \| nothing is there \| 6 \| \| Overcome this difficulty as soon as possible, let everyone's life return to normal! \| 3 \| \| Personally, I think the content of the questionnaire is quite good, very detailed and comprehensive \| 4 \| \| There's nothing left \| 3 \| |
| --- | --- | --- | --- | --- | --- | --- | --- | --- | --- | --- | --- | --- | --- | --- | --- | --- | --- | --- | --- | --- | --- | --- | --- | --- | --- | --- | --- | --- | --- | --- | --- | --- | --- | --- | --- | --- | --- | --- | --- | --- | --- | --- | --- | --- | --- | --- | --- | --- |

**Table S2.** Comparison of key study variables between the valid set and the fraud set produced by our multilayer fraud detection methods. P values from chi-squared tests for categorical variables, t-tests for normally distributed continuous variables, and Mann-Whitney U tests for continuous variables that were not normally distributed.

|  | Multilayer Valid Set (n=3228) | Multilayer Fraud Set (n=4722) | p value |  |
| --- | --- | --- | --- | --- |
| ***Demographics*** |  |  |  |  |
| Age (mean (SD)) | 38.09 (12.15) | 33.80 (7.08) | <0.001 | |
| Race/ethnicity (%) |  |  | <0.001 | |
| Hispanic/Latinx | 254 ( 7.9) | 934 (19.8) |  | |
| NH^1^-Multiracial/Other | 120 ( 3.7) | 26 ( 0.6) |  | |
| NH^1^-American Indian or Alaska Native | 7 ( 0.2) | 128 ( 2.7) |  | |
| NH^1^-Asian | 219 ( 6.8) | 92 ( 1.9) |  | |
| NH^1^-Black or African American | 728 (22.6) | 1128 (23.9) |  | |
| NH^1^-Native Hawaiian/Pacific Islander | 11 ( 0.3) | 31 ( 0.7) |  | |
| NH^1^-White | 1889 (58.5) | 2383 (50.5) |  | |
| Gender (%) |  |  | <0.001 | |
| Woman | 2028 (62.8) | 2225 (47.1) |  | |
| Man | 1108 (34.3) | 2463 (52.2) |  | |
| Transgender or gender diverse | 76 ( 2.4) | 29 ( 0.6) |  | |
| Prefer not to answer | 16 ( 0.5) | 5 ( 0.1) |  | |
| Sexual Orientation (%) |  |  | <0.001 | |
| Bisexual | 242 ( 7.5) | 119 ( 2.5) |  | |
| Gay | 101 ( 3.1) | 130 ( 2.8) |  | |
| Lesbian | 69 ( 2.1) | 73 ( 1.5) |  | |
| Straight (i.e., not gay, lesbian or bisexual) | 2682 (83.1) | 4357 (92.3) |  | |
| Other | 84 ( 2.6) | 10 ( 0.2) |  | |
| Prefer not to answer | 50 ( 1.5) | 33 ( 0.7) |  | |
| Education (%) |  |  | <0.001 | |
| Less than high school | 49 ( 1.5) | 166 ( 3.5) |  | |
| High school or equivalent | 299 ( 9.3) | 714 (15.1) |  | |
| Some college | 579 (17.9) | 1311 (27.8) |  | |
| College graduate | 1672 (51.8) | 2263 (47.9) |  | |
| Graduate degree | 620 (19.2) | 262 ( 5.5) |  | |
| Prefer not to answer | 9 ( 0.3) | 6 ( 0.1) |  | |
| Survey Type (%) |  |  | <0.001 | |
| Adult | 1070 (33.1) | 473 (10.0) |  | |
| Parent | 1812 (56.1) | 4032 (85.4) |  | |
| Youth | 346 (10.7) | 217 ( 4.6) |  | |
|  |  |  |  | |
| ***Survey metrics*** |  |  |  | |
| Survey Duration, minutes (median [IQR]) | 22.02 [18.13, 32.57] | 25.00 [18.67, 41.20] | <0.001 | |
| User Language = Spanish (%) | 22 ( 0.7) | 105 ( 2.2) | <0.001 | |
|  |  |  |  | |
| ***Key study variables*** |  |  |  | |
| Ever tested for COVID-19 = Yes (%) | 2840 (88.0) | 4128 (87.4)*NS | 0.478 | |
| Ever COVID-19 positive (%) |  |  | <0.001 | |
| No | 2498 (87.3) | 3338 (80.1) |  | |
| Yes | 352 (12.3) | 746 (17.9) |  | |
| Don’t know/ Prefer not to answer | 10 ( 0.3) | 85 ( 2.0) |  | |
| NA | 368 (11.4) | 553 (11.7) |  | |
| COVID-19 Vaccination Status (%) |  |  | <0.001 | |
| No, have not gotten the vaccine | 66 ( 2.0) | 392 ( 8.3) |  | |
| Yes, first dose of two-dose vaccine | 137 ( 4.2) | 628 (13.3) |  | |
| Yes, both doses of two-dose vaccine | 2815 (87.2) | 2703 (57.2) |  | |
| Yes, one-dose vaccine | 166 ( 5.1) | 767 (16.2) |  | |
| Yes, more than two doses of a vaccine | 32 ( 1.0) | 164 ( 3.5) |  | |
| Don’t know/ Prefer not to answer | 12 ( 0.4) | 68 ( 1.4) |  | |
| COVID-19 Vaccine Confidence (%) |  |  | <0.001 | |
| Not at all confident | 43 ( 1.3) | 207 ( 4.4) |  | |
| Not too confident | 399 (12.4) | 965 (20.4) |  | |
| Somewhat confident | 1055 (32.7) | 1986 (42.1) |  | |
| Very confident | 1696 (52.5) | 1488 (31.5) |  | |
| Don’t know/ Prefer not to answer | 35 ( 1.0) | 76 ( 1.6) |  | |

^1^NH - non-Hispanic

**Table S3.** Comparison of key study variables between the valid set and the fraud set produced by the Qualtrics fraud detection methods. P values from chi-squared tests for categorical variables, t-tests for normally distributed continuous variables, and Mann-Whitney U tests for continuous variables that were not normally distributed.

|  | Qualtrics Valid Set (n=4389) | Qualtrics Fraud Set (n=3561) | p value |
| --- | --- | --- | --- |
| ***Demographics*** |  |  |  |
| Age (mean (SD)) | 37.01 (10.81) | 33.73 (7.76) | <0.001 |
| Race/ethnicity (%) |  |  | <0.001 |
| Hispanic/Latinx | 571 (13.0) | 617 (17.3) |  |
| NH^1^-Multiracial/Other | 100 ( 2.3) | 46 ( 1.3) |  |
| NH^1^-American Indian or Alaska Native | 34 ( 0.8) | 101 ( 2.8) |  |
| NH^1^-Asian | 221 ( 5.0) | 90 ( 2.5) |  |
| NH^1^-Black or African American | 853 (19.4) | 1003 (28.2) |  |
| NH^1^-Native Hawaiian/Pacific Islander | 10 ( 0.2) | 32 ( 0.9) |  |
| NH^1^-White | 2600 (59.2) | 1672 (47.0) |  |
| Gender (%) |  |  | <0.001 |
| Woman | 2645 (60.3) | 1608 (45.2) |  |
| Man | 1663 (37.9) | 1908 (53.6) |  |
| Transgender or gender diverse | 64 ( 1.5) | 41 ( 1.2) |  |
| Prefer not to answer | 17 ( 0.4) | 4 ( 0.1) |  |
| Sexual Orientation (%) |  |  | <0.001 |
| Bisexual | 262 ( 6.0) | 99 ( 2.8) |  |
| Gay | 147 ( 3.3) | 84 ( 2.4) |  |
| Lesbian | 65 ( 1.5) | 77 ( 2.2) |  |
| Straight (i.e., not gay, lesbian or bisexual) | 3791 (86.4) | 3248 (91.2) |  |
| Other | 71 ( 1.6) | 23 ( 0.6) |  |
| Prefer not to answer | 53 ( 1.2) | 30 ( 0.8) |  |
| Education (%) |  |  | <0.001 |
| Less than high school | 65 ( 1.5) | 150 ( 4.2) |  |
| High school or equivalent | 479 (10.9) | 534 (15.0) |  |
| Some college | 964 (22.0) | 926 (26.0) |  |
| College graduate | 2253 (51.3) | 1682 (47.2) |  |
| Graduate degree | 617 (14.1) | 265 ( 7.4) |  |
| Prefer not to answer | 11 ( 0.3) | 4 ( 0.1) |  |
| Survey Type (%) |  |  | <0.001 |
| Adult | 1078 (24.6) | 465 (13.1) |  |
| Parent | 2950 (67.2) | 2894 (81.3) |  |
| Youth | 361 ( 8.2) | 202 ( 5.7) |  |
|  |  |  |  |
| ***Survey metrics*** |  |  |  |
| Survey Duration, minutes (median [IQR]) | 22.82 [18.52, 35.13] | 24.78 [18.12, 40.47] | <0.001 |
| User Language = Spanish (%) | 53 ( 1.2) | 74 ( 2.1) | 0.003 |
|  |  |  |  |
| ***Key study variables*** |  |  |  |
| Ever tested for COVID-19 = Yes (%) | 3903 (88.9) | 3065 (86.1) | <0.001 |
| Ever COVID-19 positive (%) |  |  | 0.011 |
| No | 3294 (83.8) | 2542 (82.0) |  |
| Yes | 593 (15.1) | 505 (16.3) |  |
| Don’t know/ Prefer not to answer | 42 ( 1.1) | 53 ( 1.7) |  |
| NA | 460 (10.5) | 461 (12.9) |  |
| COVID-19 Vaccination Status (%) |  |  | <0.001 |
| No, have not gotten the vaccine | 215 ( 4.9) | 243 ( 6.8) |  |
| Yes, first dose of two-dose vaccine | 287 ( 6.5) | 478 (13.4) |  |
| Yes, both doses of two-dose vaccine | 3465 (78.9) | 2053 (57.7) |  |
| Yes, one-dose vaccine | 295 ( 6.7) | 638 (17.9) |  |
| Yes, more than two doses of a vaccine | 111 ( 2.5) | 85 ( 2.4) |  |
| Don’t know/ Prefer not to answer | 16 ( 0.3) | 64 ( 1.8) |  |
| COVID-19 Vaccine Confidence (%) |  |  | <0.001 |
| Not at all confident | 144 ( 3.3) | 106 ( 3.0) |  |
| Not too confident | 732 (16.7) | 632 (17.7) |  |
| Somewhat confident | 1558 (35.5) | 1483 (41.6) |  |
| Very confident | 1898 (43.2) | 1286 (36.1) |  |
| Don’t know/ Prefer not to answer | 57 ( 1.3) | 54 ( 1.5) |  |

^1^NH - non-Hispanic
